# Proteomic and transcriptomic profiling of aerial organ development in Arabidopsis

**DOI:** 10.1038/s41597-020-00678-w

**Published:** 2020-10-09

**Authors:** Julia Mergner, Martin Frejno, Maxim Messerer, Daniel Lang, Patroklos Samaras, Mathias Wilhelm, Klaus F. X. Mayer, Claus Schwechheimer, Bernhard Kuster

**Affiliations:** 1 Chair of Proteomics and Bioanalytics, Technical University of Munich (TUM), Freising, Germany; 2 Plant Genome and Systems Biology, Helmholtz Center Munich, German Research Center for Environmental Health, Munich-Neuherberg, Germany; 3 Plant Genome Biology, Technical University of Munich (TUM), Freising, Germany; 4 Chair of Plant Systems Biology, Technical University of Munich (TUM), Freising, Germany; 5 Bavarian Center for Biomolecular Mass Spectrometry (BayBioMS), TUM, Freising, Germany

**Keywords:** Plant morphogenesis, Proteomic analysis, Arabidopsis thaliana

## Abstract

Plant growth and development are regulated by a tightly controlled interplay between cell division, cell expansion and cell differentiation during the entire plant life cycle from seed germination to maturity and seed propagation. To explore some of the underlying molecular mechanisms in more detail, we selected different aerial tissue types of the model plant *Arabidopsis thaliana*, namely rosette leaf, flower and silique/seed and performed proteomic, phosphoproteomic and transcriptomic analyses of sequential growth stages using tandem mass tag-based mass spectrometry and RNA sequencing. With this exploratory multi-omics dataset, development dynamics of photosynthetic tissues can be investigated from different angles. As expected, we found progressive global expression changes between growth stages for all three omics types and often but not always corresponding expression patterns for individual genes on transcript, protein and phosphorylation site level. The biggest difference between proteomic- and transcriptomic-based expression information could be observed for seed samples. Proteomic and transcriptomic data is available via ProteomeXchange and ArrayExpress with the respective identifiers PXD018814 and E-MTAB-7978.

## Abstract (summary)

**Table:** 

Measurement(s)	transcriptome • Proteome • Phosphoproteome
Technology Type(s)	RNA sequencing • liquid chromatography-tandem mass spectrometry
Factor Type(s)	organism part • growth stage
Sample Characteristic - Organism	Arabidopsis thaliana

Machine-accessible metadata file describing the reported data: 10.6084/m9.figshare.12998993

## Background & Summary

Developmental processes modulate the size, shape and functionality of an organism during its life cycle^1^. The boundaries and timelines for development are defined by the genetic code stored in the DNA complement of each cell, but how the genetic programme is executed depends on environmental conditions^2^. The model plant *Arabidopsis thaliana* is commonly used as a reference model to study many aspects of plant growth and development^3^. Arabidopsis has a short life cycle of about six weeks, which starts with seed germination and vegetative growth followed by the transition to flowering, seed production and finally seed maturation^3^. In 2001, Boyes *et al*. introduced specific growth stage definitions, which now serve as landmarks in the dynamic process of Arabidopsis development^1^. Within this uniform framework, researchers are able to compare data from phenotypic studies with metabolic and gene expression profiling data of the respective developmental growth stages.

Expression profiling generates a link between gene information and tissue morphogenesis or plant phenotype. Transcriptomic analyses are commonly used to study the regulation of growth and development. However, as proteins are the executers of most response programs, a combined transcriptomic and proteomic approach should enable deeper insights into the molecular changes during plant development. Work from this laboratory has recently provided such a combined study and assembled a mass spectrometric draft of the proteome of Arabidopsis^4^. This has provided important clues as to which Arabidopsis genes exist as proteins, where they are found within the organism and in which approximate quantities. While very powerful as such, this resource portraits merely a static picture. Clearly, having both spatial (different tissue types) and temporal (growth stages) information is often necessary to elucidate gene functions involved in the dynamic processes of plant development^5^. Advances in proteomics analysis techniques via liquid chromatography coupled to tandem mass spectrometry now allow sampling of a proteome to an unprecedented depth but often require extensive sample fractionation steps^6^. In addition to substantially increasing data acquisition time, this leads to increased quantitative variability and reduced data completeness between experiments. Stable isotope labelling approaches like tandem mass tags (TMT) allow multiplexing of up to 16 samples^7,8^ and thus enable the simultaneous measurement of samples collected e. g. over time. We choose TMT-based quantification to ensure consistent protein and phosphorylation site quantification between growth stages and to minimize the number of missing values especially in the phosphorylation data, where quantifications are mostly based on a single peptide^9^. For our study of expression profiles in consecutive developmental stages in Arabidopsis, we decided to focus on leaf, flower and fruit (silique/seed). With this selection we exemplify the value of longitudinal proteome profiling in three aerial plant tissues types. Note that the same approach may be taken for other plant parts such as stem or root.

Leaves are often considered the most important plant organs because of their role in energy metabolism and carbon fixation^10^. In Arabidopsis, the leaves at the base of the plant (rosette leaves) display different morphologies dependent on their respective age. The seedling and juvenile-phase leaves are small, round and without leaf hairs while the adult phase leaves are large and narrow, with more serrations and leaf hairs on both the upper and lower side^10^. Because rosette leaves are generated consecutively in a spiral pattern by the shoot apical meristem, juvenile stage leaves are chronologically older than adult leaves^11^. Rosette growth is still a part of the vegetative phase of Arabidopsis development and the transition to the reproductive phase starts with the onset of flowering^1^. Flowers are the most specialized organs in Arabidopsis and consist of four different organ types^12^. The green sepals, the white petals, the stamen containing the pollen with the male gametophyte and the carpel containing the female egg cells. Throughout flower development, these organ types undergo both morphological changes and growth, ending with fertilization of the egg-cell and the subsequent start of embryogenesis^12^. Like the other developmental steps, embryogenesis and seed generation are continuous processes, but can be separated into early stages, determined by pattern formation and morphogenesis, followed by maturation and the building of storage reserves in the mid phase and finally the preparation for desiccation and developmental arrest in the late stage^13^.

For our analysis, we used TMT multiplexing of consecutive rosette leaf, flower and siliques/seed stages, to generate proteomic and phosphoproteomic profiles of their expression patterns. In addition, we used RNAseq to provide the matching transcriptome dataset. Together, these three datasets provide detailed spatial and temporal information about important aspects of plant development across multiple omics dimensions and can be used as a reference dataset or hypothesis generator for future biological experiments.

## Methods

### Plant materials and growth conditions

*Arabidopsis thaliana* wild type Columbia-0 (Col-0) plants were grown on soil under continuous white light conditions at 22 °C. Samples for flower (stage 9–15), siliques and seeds were harvested from mature plants. Seed stages were collected from developing siliques and processed either with (silique, stage 1–5) or without (seed, stage 6–10) silique septum and valves. Juvenile and adult rosette leaves were harvested at the same time point from 22 days-old plants before bolting. Classification of growth stage and plant section was done as described before^12–14^. Harvested material from at least three individual plants was combined for each sample, frozen in liquid nitrogen and stored at −80 °C until further use.

### Protein lysis and digest

**Fig. 1:**
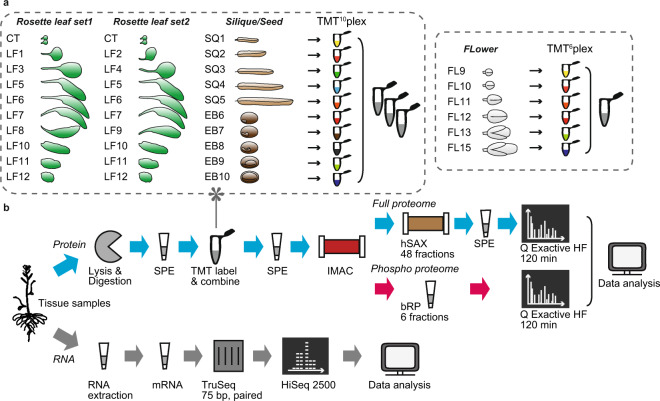
Sample description and experimental workflow. (**a**) Selected tissue growth stages and tandem mass tag (TMT) labelling scheme for rosette leaf, flower and silique/seed samples. Rosette leaf and seed/silique samples were labelled with TMT10plex and flower samples with TMT6plex reagents. Cotyledons (CT), leaf (LF), silique (SQ), seed (EB), flower (FL). (**b**) Schematic depiction of the proteomic and RNAseq workflows. Solid phase extraction (SPE), ion metal affinity chromatography (IMAC), hydrophilic strong anion exchange chromatography (hSAX), basic reversed phase chromatography (bRP).

Frozen plant material was homogenized with a tissue lyzer (Qiagen, Hilden, Germany) or with mortar and pestle in liquid nitrogen. Proteins were precipitated over night with 10% trichloroacetic acid in acetone at −20 °C and subsequently washed two times with ice-cold acetone. Dry samples were incubated with urea digestion buffer (8 M urea, 50 mM Tris-HCl pH 7.5, 1 mM DTT, cOmplete^TM^ EDTA-free protease inhibitor cocktail (PIC) [Roche, Basel, Switzerland], Phosphatase inhibitor [PI-III; in-house, composition resembling Phosphatase inhibitor cocktail 1,2 and 3 from Sigma-Aldrich, St. Louis, USA]) for 1 h. Protein concentration was determined with a Bradford assay^15^. For each sample 100 µg (TMT10plex) or 166 ug (TMT6plex) of protein was reduced (10 mM DTT), alkylated (55 mM chloroacetamide), and diluted 1:8 with digestion buffer (50 mM Tris-HCl pH 8.0, 1 mM CaCl_2_). In-solution digestion with trypsin (1:100 w/w) (Roche, Basel, Switzerland) at 37 °C was performed for 4 h followed by a second digestion step over night. Digested samples were acidified to pH 3 using trifluoroacetic acid (TFA) and centrifuged at 14,000 *g* for 15 min at 4 °C. The supernatants were desalted on 50 mg SepPAC columns (Waters, Milford, USA) and vacuum dried. TMT labelling was performed as described previously^16,17^. To cover the 13 rosette leaf series samples, two separate TMT10plex experiments were performed with seven leaf stages as biological replicates (CT, LF5-6, LF10-12) and either LF1,3,8 (set 1) or LF2,4,9 (set 2) as variable subsets (Fig. 1a).

### Peptide enrichment and fractionation

Fe^3+^-IMAC was performed as described previously with some adjustments^18^. TMT-labelled peptides of the growth stage samples were combined for each respective developmental series, desalted, vacuum dried and subsequently re-suspended in ice-cold IMAC loading buffer (0.1% TFA, 40% acetonitrile). For quality control, 1.5 nmol of a synthetic library of phosphopeptides and their corresponding non-phosphorylated counterpart sequence (B2 and F1)^19^ were spiked into each sample prior to loading onto a Fe^3+^-IMAC column (Propac IMAC-10 4 × 50 mm, Thermo Fisher Scientific, Waltham, USA). The enrichment was performed with Buffer A (0.07% TFA, 30% acetonitrile) as wash buffer and Buffer B (0.315% NH_4_OH) as elution buffer. Collected full proteome and phosphopeptide fractions were vacuum-dried and stored at −80 °C until further use.

For the full proteome analysis, hydrophilic strong anion exchange chromatography (hSAX) peptide separation was performed as described previously^20^. The full proteome IMAC fraction was reconstituted in hSAX solvent A (5 mM Tris-HCl, pH 8.5) and an equivalent of 300 µg protein digest separated using a Dionex Ultimate 3000 HPLC system (Dionex Cor., Idstein, Germany) equipped with an IonPac AG24 guard column (2 × 50 mm) and an IonPac AS24 stong anion exchange column (2 × 250 mm, Thermo Fisher Scientific, Waltham, USA). Fractions were collected in 96 well format and subsequently pooled to 48. Individual fractions were acidified with formic acid (FA), desalted on self-packed StageTips (five disks, Ø 1.5 mm C18 material, 3 M Empore^TM^, elution solvent 0.1% FA in 50% ACN) and dried down prior to LC-MS/MS analysis. Phosphopeptides were fractionated into six fractions using the high pH reversed phase protocol and pooling scheme for TMT-labelled phosphopeptides as described previously^16^. Phosphopeptide fractions were reconstituted in desalting buffer (0.1% FA) and loaded on self-packed StageTips (five disks, Ø 1.5 mm C18 material, 3 M Empore^TM^). After a wash step with desalting buffer, basic reversed phase buffer A (25 mM NH_4_FA pH 10) was applied to the StageTips and the flow through (FT) collected in a new vial. Phophosphopeptides were eluted with 5%, 7.5%, 12.5%, 17.5% and 50% ACN in 25 mM NH_4_FA pH 10. The 5% and 50% ACN and the FT and 17.5% fractions were combined and all fractions were dried down prior to LC-MS/MS analysis.

### LC-MS/MS analysis

Nanoflow LC-MS/MS was performed by coupling a Dionex 3000 (Thermo Fisher Scientific, Waltham, USA) to a QExactive Orbitrap HF (Thermo Fisher Scientific, Waltham, USA). Samples for full proteome and phosphoproteome analysis were re-suspended in loading buffer containing 0.1% formic acid (FA) or 50 mM citrate and 1% FA, respectively. Peptide loading and washing were done on a trap column (100 µm i.d. x 2 cm, packed in-house with Reprosil-Pur C18-GOLD, 5 µm resin, Dr. Maisch, Ammerbuch, Germany) at a flow rate of 5 µl/min in 100% loading buffer (0.1% FA) for 10 min. Peptide separation was performed on an analytical column (75 µm i.d. x 40 cm packed in-house with Reprosil-Pur C18, 3 µm resin, Dr. Maisch, Ammerbuch, Germany) at a flow rate of 300 nl/min using a 110 min gradient from 4% to 32% solvent B (solvent A: 0.1% FA, 5% DMSO in HPLC grade water; solvent B: 0.1% FA, 5% DMSO in acetonitrile) for the full proteome analysis and a two-step 110 min gradient from 4% to 27% solvent B for the phosphoproteome analysis^21^. Peptides were ionized using a spray voltage of 2.2 kV and a capillary temperature of 275 °C. The instrument was operated in data-dependent mode, automatically switching between MS and MS2 scans. Full scan MS spectra (m/z 360–1300) were acquired with a maximum injection time of 10 ms at 60,000 resolution and an automatic gain control (AGC) target value of 3e6 charges. For the top 20 precursor ions, high resolution MS2 spectra were generated in the Orbitrap with a maximum injection time of 57 ms at 30,000 resolution (isolation window 1.3 m/z), an AGC target value of 2e5 and normalized collision energy of 33%. The underfill ratio was set to 1% with a dynamic exclusion of 20 s. Only precursors with charge states between 2 and 6 were selected for fragmentation. For the phosphoproteome analysis, the MS2 spectra were acquired with a maximum injection time of 100 ms. Dynamic exclusion was set to 35 s.

### Peptide and protein identification and quantification

Raw data files for full proteome and phosphoproteome were processed together as two separate parameter groups using MaxQuant software (v. 1.5.3.8) with standard settings unless otherwise described^22^. MS/MS spectra were searched against Araport11^23^ protein coding genes (Araport11_genes.201606.pep.fasta; download 06/2016), known contaminants and spike-in phosphopeptide library sequences^19^, with trypsin as protease and up to two allowed missed cleavages. Carbamidomethylation of cysteines was set as fixed modification and oxidation of methionines and N-terminal acetylation as variable modifications. For the phosphoproteome parameter group phosphorylation of serine, threonine or tyrosine was added as variable modification. Search parameters for the TMT-labelled full- and phosphoproteome were adjusted according to TMT6 plex/10plex settings (PIF > 0.75, TMT batch correction factors). Results were filtered to 1% PSM, protein and Site FDR.

### RNA sequencing

Total RNA was isolated using the NucleoSpin RNA Plant kit (Macherey-Nagel, Düren, Germany). DNA was removed by on-column treatment with rDNAse (Macherey-Nagel, Düren, Germany). For recalcitrant samples (seed, silique), a LiCl-based protocol was adopted with minor modifications^24^. After LiCl precipitation, the RNA pellet was dissolved in rDNAse buffer and treated with rDNAse (Macherey-Nagel, Düren, Germany) at 37 °C for 10 min. The final pellet was re-suspended in 35 µl DEPC-treated water. RNA was quantified (Nanodrop^TM^, Thermo Fisher Scientific, Waltham, USA) and quality checked with a Bioanalyzer 2100 (Agilent Technologies, Santa Clara, USA). RNA integrity number (RIN) values between 6.4 and 10 were accepted for further analysis. cDNA libraries were prepared using the TruSeq Stranded mRNA Sample Preparation kit (Illumina, San Diego, USA) according to the manufacturer’s instructions. Clusters were generated in two batches and sequenced on a High throughput flow cell with the HiSeq. 2500 platform (Illumina, San Diego, USA) to a depth of 36 million reads per sample. Quality assessment of raw and trimmed 75 bp paired RNAseq reads was performed with FastQC. Raw RNAseq reads were trimmed to remove adapter contamination and poor quality base calls using Trimmomatic version 0.35 with parameters (ILLUMINACLIP:Illumina-PE.fasta:2:30:10; LEADING:3; TRAILING:3; SLIDINGWINDOW:4:20; MINLEN:36)^25^. Trimmed RNAseq reads were mapped to the Araport11^23^ transcriptome with Kallisto version 0.43.1 (default parameters)^26^.

### Data processing

MaxQuant output tables were filtered for non-plant contaminants, reversed sequences and proteins which were only identified based on modified peptides. Protein abundance estimation was based on corrected TMT reporter intensities. For comparison of genes identified at transcript and protein levels, MaxQuant ProteinGroups containing several gene loci were filtered out in order to retain only unambiguously identified gene loci. In case of multiple protein isoform identifications as distinct ProteinGroups, only the isoform with the higher number of razor + unique peptides was retained. For qualitative and quantitative analyses, all protein or transcript isoform information was collapsed onto the gene level. Note therefore, that we use the term protein identification to describe the identification of specific gene locus with at least one peptide and do not consider the various proteoforms this might contain. mRNA quantities are displayed as transcripts per kilobase million (TPM) and a cutoff of 1 TPM was used as lower limit for detection across all samples.

Unless otherwise stated, displayed abundances for protein, transcript and phosphorylation sites were log_2_ transformed. Protein and transcript datasets were median centred to the overall median of the respective dataset. No normalization was performed for the p-site dataset, since total p-site intensity variations between tissues are also due to biological sample differences. Instead, the spike-in phosphopeptide library was used, to assess reproducible enrichment efficiency and MS measurement quality of phosphoproteome samples^19^. Phosphoproteins were defined as proteins with a distinct phosphorylation site identification. P-sites with a localization probability >0.75 were designated as class I sites^27^. ComBat^28^ was used to remove batch effects between the two TMT10plex experiments covering the 13 rosette leaf samples after log transformation and mean abundance calculation of protein/p-sites between replicate samples.

### Data analysis

Araport11 annotated Arabidopsis gene loci (n = 27,655) and Isoform (n = 48,359) coverage was calculated using all transcript identifications and unambiguously identified proteins. Note that only 35,870 isoforms have a distinct sequence on protein level. Histograms of the log_2_ transformed transcript abundance distribution were plotted in Perseus^29^ (v. 1.5.5.3) and the population of transcripts that was also identified on protein level indicated for each tissue type.

Pearson correlation coefficients of protein TMT reporter intensities between different tissue growth stages were calculated in Perseus using ProteinGroups with unambiguous gene loci identification and at least one valid quantification (LF n = 9,080; FL n = 9,706; SQ/EB n = 11,276). For the two rosette leaf TMT experiments, Pearson correlation coefficients were calculated for all pair-wise combinations of TMT reporter intensities of growth stages from leaf set 1 and leaf set 2. Pearson correlation coefficients of transcript intensities between different tissue growth stages were calculated using all transcripts with at least one quantification (LF n = 19,759; FL n = 22,632; SQ/EB n = 22,506).

Principal component analysis (PCA) was performed in Perseus using z-scored protein and transcript intensities and datasets without missing values for both omics levels (LF n = 7,563; FL n = 9,138; SQ/EB n = 9,559)

Supervised hierarchical clustering analysis on protein level (unambiguous identifications) for consecutive developmental stages (rosette leaf, flower; silique, seed) was performed on log_2_-transformed, z-scored intensities in Perseus using Euclidean distance and average linkage. Z-scoring was performed separately for silique and seed samples because the large morphological difference between the two sample sets would overshadow small changes between growth stages. Gene ontology biological process (GOBP) term annotations were loaded from the Perseus organism repository (mainAnnot.arabidopsis_thaliana.txt; download 10/2015). A Fisher’s exact test was performed for the protein expression clusters in each dataset using Benjamini-Hochberg FDR truncation (0.01 threshold). The results were filtered for an enrichment factor > 1.5 (Supplemental Tables S1–3).The relation between protein and transcript expression for individual gene loci was calculated using the Pearson correlation coefficient and the set of genes with abundance measurements on both protein and transcript level in at least five matching growth stages (5 pairwise complete observations; LF n = 8,938; FL n = 9,268; SQ n = 10,568; EB n = 9,868). Density distributions of Pearson correlation values were plotted in R^30^ (v 3.5.1). To estimate the relative proportion of genes with positive (Pearson coefficient: 0.5 to 1), negative (Pearson coefficient: −1 to −0.5) and no correlation (Pearson coefficient:−0.5 to 0 and 0 to 0.5) between transcript and protein levels for each dataset, the number of genes in each section was divided by the total number of genes in each dataset. Supervised hierarchical clustering analysis was performed in Perseus for genes with Pearson correlation coefficient > 0.5 (n = 4,264) or < −0.5 (n = 1,724) in the seed dataset using log_2_-transformed and z-scored intensities, Euclidean distance and average linkage. A Fisher’s exact test was performed in Perseus for the expression clusters in both categories (positive protein-transcript correlation + ; negative protein-transcript correlation –) using Benjamini-Hochberg FDR truncation (Supplemental Table S3). Enrichment factor, the negative logarithm of the Benjamini-Hochberg FDR and category size were plotted for GOBP categories that passed the 0.01 FDR threshold.

Domain information for IQD14 (AT2G43680) was obtained from the pfam database^31^ (http://pfam.xfam.org/protein/Q8LPG9) for the identifier IQD14_ARATH. We note that with the exception of the leaf dataset (n = 2 for 7 stages), no replicates were performed for the developmental time courses and the dataset should therefore be treated as exploratory. Similar developmental stages however show high expression similarity and can be used to contrast e.g. early and late development stages.

## Data Records

Transcriptome sequencing and quantification data are available at ArrayExpress (www.ebi.ac.uk/arrayexpress) under the identifier E-MTAB-7978^32^. The raw mass spectrometric data and MaxQuant result files have been deposited to the ProteomeXchange Consortium via PRIDE^33^, with the dataset identifier PXD018814^34^. The datasets will also be available via ProteomicsDB^35^.

## Technical Validation

### Experimental design

High quality data and a good coverage of the transcriptome/proteome are essential to gain meaningful information about the function of biological pathways and individual genes throughout plant growth stages. For our expression profiling of different stages in Arabidopsis growth, we placed a focus on four aerial organ systems, rosette leaves, siliques, immature seeds and flowers and collected samples spanning a defined segment in their respective development (Fig. 1a). To cover the 13 samples of the rosette leaf series with the available ten TMT isobaric labelling channels, we performed two independent proteomics experiments. Cotyledons and rosette leaves number 5, 6, 7, 10, 11 and 12 are represented in both experiments as biological replicates. The leaves 1 and 2 (juvenile phase), 3 and 4 (juvenile phase) and 8 and 9 (adult phase) respectively, are morphological very similar and were divided between the two leaf datasets. Leaves 1, 3 and 8 were covered in the first leaf series experiment, leaves 2, 4 and 9 in the second (Fig. 1a). We used isobaric labelling with TMT and extensive peptide fractionation in combination with measurement on a Q Exactive HF mass spectrometry platform to obtain deep and consistent proteome coverage and quantification (Fig. 1b). Part of the protein samples were used to generate sample-matched profiles of the phosphorylation status across organ development. The enrichment of phosphopeptides in each series was performed after the TMT labelling step to reduce technical variance (see methods; Fig. 1b). In parallel, we also extracted total RNA from all leaf, silique, seed and flower stages and measured transcript abundance profiles by RNAseq using a HiSeq. 2500 sequencer (Fig. 1b).

### Qualitative and quantitative transcriptome and proteome coverage

**Fig. 2:**
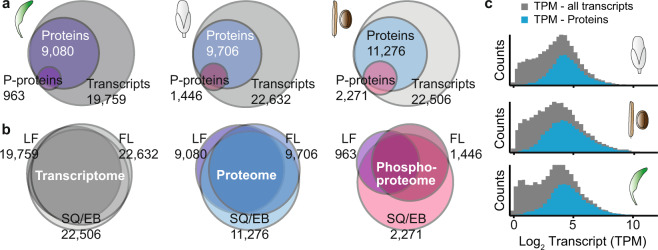
Number of identifications across samples. (**a**) Overlap between Arabidopsis protein coding genes identified on transcript, protein and phosphoprotein level for rosette leaf (left panel), flower (middle panel) and the silique/seed (right panel). (**b**) Overlap between Arabidopsis protein coding genes identified in the different tissue datasets on transcript (left panel), protein (middle panel) and phosphoprotein level (right panel). (**c**) TPM intensity distribution of transcripts identified in this study. The number of transcripts also identified as proteins are highlighted in blue.

Using the above described workflow, we identified more than 9,000 distinct proteins for each organ type (Table 1; Fig. 2a; Supplemental Tables S1–3). Protein identifications in silique/seed and flower were higher than in leaves. This can be explained by the high dynamic range of photosynthesis-associated proteins in leaves that mask the presence of more low abundant proteins^4,36^ (Fig. 2b). In addition, we expect a more varied gene expression in flower and silique/seed tissues given their specialized cell types and morphology. Silique and seed tissues showed the highest phosphorylation activity with the largest number of identified phosphorylation sites (Fig. 2b). The average sequence coverage was 20.1% for flower, 27.8% for silique/seed and 25.6% for the leaf dataset, respectively (Table 2) and falls within the expected range for tryptic digested samples. More than 90% of the proteins in each dataset were identified with two or more unique peptides and the average Andromeda score (all peptides, Table 2) was 64.2, 95.6 and 103.0, respectively. In the parallel RNAseq analysis we identified up to 22,632 individual transcripts thus covering about 82% (flower) of the annotated protein-coding genes (Araprot11^23^) which is about twice as high as coverage on the proteome level (Table 3). Due to the lower sequence coverage in proteomics, isoform identification is also more effective on transcript than on protein level (Table 3). Note that nearly all protein identifications arise from the high abundant transcript populations (Fig. 2c), which shows the current limitation of the proteomics technology to detect low abundant protein species in the background of highly complex samples. With the ongoing advances in mass spectrometry technology and bioinformatics tools however, we expect to see even more sensitive MS analyses in the future.

**Table 1:** Number of identifications on protein, transcript and phosphorylation level.

Identification	Rosette leaf	Flower	Silique/Seed
ProteinGroups (all)	10,123	10,686	12,225
ProteinGroups (unambiguous)	9,080	9,706	11,276
Phosphoproteins (unambiguous)	963	1,446	2,271
Peptides (all)	87,364	68,011	105,469
Phosphopeptides (all)	1,576	2,299	4,297
Phosphorylation sites (all)	1,838	2,596	4,870
Phosphorylation sites class I (all)	1,559	2,229	4,097
Transcripts	19,759	22,632	22,506

**Table 2:** Sequence coverage, unique peptide identifications and Andromeda Score.

Description	Rosette leaf	Flower	Silique/Seed
**ProteinGroups (unambiguous)**			
Sequence coverage 0%-25%	53.7%	67.4%	48.9%
Sequence coverage 25%-50%	38.3%	28.6%	40.1%
Sequence coverage 50%-75%	7.6%	3.7%	10.5%
Sequence coverage 75%-100%	0.4%	0.2%	0.6%
**ProteinGroups (unambiguous)**			
Unique peptides < 2	5.7%	8.5%	6.3%
Unique peptides 2	9.3%	14.4%	9.5%
Unique peptides > 2	85.0%	77.1%	84.2%
**Peptides (all)**			
Andromeda Score < 50	7.0%	34.4%	17.2%
Andromeda Score 50–100	39.2%	49.0%	35.8%
Andromeda Score ≥ 100	53.8%	16.6%	47.0%

**Table 3:** Percentage of protein coding gene loci (Araport11 annotation) detected on protein and transcript level.

Description	Omics Level	Rosette leaf	Flower	Silique/Seed
**Gene loci n = 27,655**				
Gene loci	Proteomics	32.8%	35.1%	40.8%
Gene loci	Transcriptomics	71.4%	81.8%	81.4%
**Isoforms n = 48,359**				
Isoforms	Proteomics	12.8%	13.1%	15.8%
Isoforms	Transcriptomics	89.2%	94.4%	93.9%

**Fig. 3:**
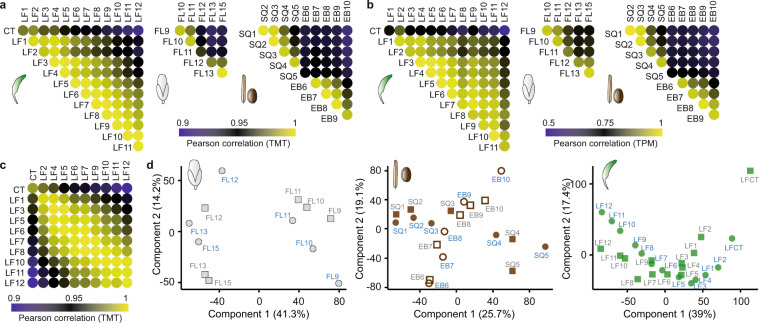
Expression profile correlation across developmental stages. (**a**) Pearson correlation between protein intensity values for the rosette leaf (left panel), the flower (middle panel) and the seed/silique (right panel) growth stages. (**b**) Pearson correlation between transcript intensity values for the rosette leaf (left panel), the flower (middle panel) and the seed/silique (right panel) growth stages. (**c**) Pearson correlation between protein intensity values of rosette leaf series one and two with biological replicates of cotyledons and rosette leaves 5, 6, 7, 10, 11 and 12. Batch effect differences between TMT experiments were adjusted with mCombat (see methods). (**d**) Principal component analysis of the proteins and transcripts identified in all samples of a tissue dataset on both transcript (squares) and protein level (circles) (LF n = 7,563; FL n = 9,138; SQ/EB n = 9,559). Proteome sample stage names are given in blue, transcriptome samples in grey.

As expected, quantitative expression levels of most proteins were very similar between consecutive growth stages but diverged during developmental progression of the respective tissues (Fig. 3a). The same was observed for quantitative expression levels of transcripts (Fig. 3b). For the rosette leaf dataset, we compared protein identifications and quantifications between the two independent biological replicates to estimate workflow variation. Protein expression level quantification was very reproducible and matching leaves and adjacent growth stages showed the best correlation (Fig. 3c). The overlap in protein identification for the two leaf TMT datasets was 89% which also demonstrated the consistency in measurement depth for one tissue type.

### Stage-specific gene expression

**Fig. 4:**
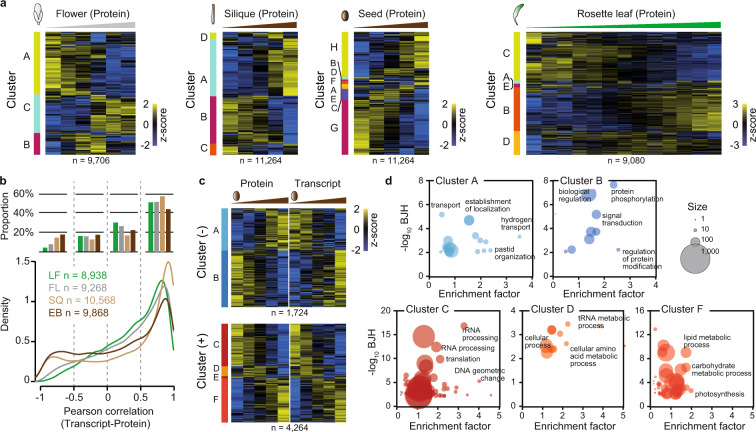
GO enrichment and protein-transcript correlation. (**a**) Supervised hierarchical clustering of z-scored gene expression profiles at protein level for leaf, flower, silique and seed datasets. (**b**) Density profiles of Pearson correlation coefficients between transcript and protein abundance for individual genes (lower panel). Bar chart displaying the respective proportion of genes with positive (0.5 to 1), negative (−1 to −0.5) or no correlation (−0.5 to 0 and 0 to 0.5) (upper panel). Rosette leaf (LF), flower (FL), silique (SQ) and seed (EB). (**c**) Supervised hierarchical clustering of z-scored gene expression profiles at protein and transcript level for genes with positive (+; 0.5 to 1) or negative (-; -1 to −0.5) protein-transcript correlation. (**d**) Fisher’s exact test GO term enrichment for clusters from (**c**). Benjamini-Hochberg (BJH) threshold 0.01.

Principal component analysis of the consecutive samples in each tissue type showed a gradual shift of expression levels from early to late developmental stages both on protein and transcript level (Fig. 3d). We expect that the comparison of gene expression profiles on the different omics levels of this dataset will allow for a more comprehensive molecular characterization of specific growth regulation^37,38^. The observed expression dynamics likely reflect a combination of tissue composition and functionality change^39^. Early and late developmental stages in the different tissues were consistently associated with specific GOBP terms. In young stages of flower (FL9,10,11), silique (SQ1,2,3) and seed (EB6,7,8) as well as newly generated rosette leaves (LF10-12) genes involved in translation, RNA processing and DNA organization are comparatively high abundant as would be expected of tissues with a high amount of cell division activity (Fig. 4a; Supplemental Tables S1–3). Later stages on the other hand are dominated by energy generation, transport and metabolic processes (Fig. 4a; Supplemental Tables S1–3).

## Usage Notes

With the following examples we aim to exemplify, how the information provided in our multi-omics study can be used to explore molecular pathways that are modulated during the growth of photosynthetic active aerial plant organs.

### Protein/mRNA relation

Changes in transcript abundance are usually reflected by protein level changes in the same direction albeit not necessary of comparable magnitude^40^. We compared transcript and protein expression patterns across growth stages in our datasets and found positive correlations for a majority of genes (Fig. 4b). The seed dataset however showed an increase in the proportion of genes with either no or even negative correlation between protein and transcript levels (Fig. 4b). A possible explanation is the accumulation of storage reserves as either mRNAs or proteins that takes place during seed maturation^41^, an effect we already observed in our tissue atlas study^4^. A GOBP term enrichment analysis for the seed sample indeed showed an increase in genes associated with transport, localization and biological signalling for genes with negative protein-transcript correlations (Fig. 4c,d; Supplemental Table S3). Genes with positive protein-transcript correlations that are high abundant in the early stages of seed development on the other hand are enriched for GO terms associated with RNA processing and translation. Genes that are more abundant in the later developmental stages both on protein and transcript level can be associated with photosynthesis, energy production and metabolic processes (Fig. 4c,d; Supplemental Table S3).

### Pathway or protein family expression profiles

**Fig. 5:**
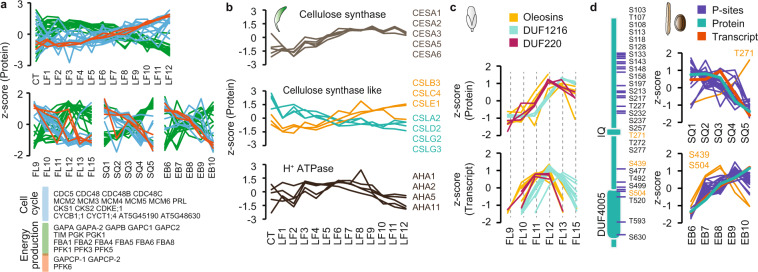
Multi-omics expression profile comparison. (**a**) Protein expression profiles for genes associated with cell cycle or energy production. (**b**) Protein expression profiles for cellulose synthase, cellulose synthase-like and H^+^ATPase proteins (rosette leaf dataset). (**c**) Protein and transcript expression profiles for Oleosin (n = 6) and domain of unknown function (DUF) 1216 (n = 10) and DUF226 (n = 5) protein families (flower dataset). (**d**) Schematic depiction of ISQ14 protein domains and localization of phosphorylation sites identified in the silique/seed dataset (left panel). Z-scored intensity profiles for IQD14 phosphorylation sites plotted together with IQD14 expression profiles on protein and transcript level (right panel).

Changes in the expression levels of proteins or protein families between different developmental stages can be associated with their molecular function. Proteins involved in cell cycle regulation and progression like cell division cycle (CDC) 5 and CDC48, cyclins, cyclin-dependent kinases or members of the minichromosome maintenance complex (MCM) are detected with higher abundance in early developmental stages of flower, silique and seed (Fig. 5a). In the rosette leaf dataset, the adult leaves 10, 11 and 12 are the morphologically youngest stages and therefore show elevated cell cycle activity in comparison to the other leaf stages (Fig. 5a). An opposite trend can be observed for genes involved in energy production like glyceraldehyde-3-phosphate dehydrogenase (GAPDH), fructose-bisphosphate aldolase (FBA) or phosphofructokinase (PFK) gene family members (Fig. 5a). Interestingly the two GAPCP-type genes (GAPCP1 and GAPCP2) are higher abundant in young tissues, similar to the cell cycle associated genes (Fig. 5a). This expression pattern is expected since these proteins are involved in glycolytic energy production in non-green plastids^42^. A similar expression pattern was observed for PFK6, suggesting a specific function in the energy metabolism of young developmental tissue stages that has yet to be elucidated^43^.

Another example that supports our protein expression data are several protein families involved in cell wall growth and turgor establishment. Members of the CESA protein family form the cellulose synthase complex (CesA) which produces cellulose, the main load bearing component of the plant cell wall^44^. Primary cell wall formation is initiated during cell division and growing cells continuously produce new cell wall components^45^. The primary cell wall CesA complex consist of multiple copies of CESA1, CESA3 and CESA6^44^. Deposition of the more rigid secondary cell wall only starts after the cessation of cell growth to mechanically stabilize especially load bearing plant structures like the stem. Within the rosette leaves we only detected components of the primary cell wall synthesis complex and CESA protein levels were more abundant in the later fast-growing leaves, which are still undergoing active cell division (Fig. 5b). This finding is supported by our previous Arabidopsis proteome study where the secondary cell wall CesA complex was primarily detected in stem tissues but not leaves^4^. Cellulose synthase-like (CSL) proteins which also belong to the cellulose synthase superfamily have been associated with the synthesis of several β-glycan polymers^46^. Among these, CSLA2 for example has been described as a mannan und glucomannan synthase^47,48^ which have structural and storage functions in the plant cell wall. CSLC4 on the other hand can synthesize xyloglucan, a major hemicellulose in the primary plant cell wall^49^. According to their functionality in cell wall formation, we detect higher expression of CSLA2 in juvenile rosette leaves and CSLC4 in later growth stages (Fig. 5b). The different expression profiles of CSL family proteins in rosette leaves can thus be used to elucidate their function in the different steps of plant cell wall synthesis and modification (Fig. 5b). Plant tissue growth occurs through cell proliferation and cell expansion^50^. After cell proliferation has stopped, cell expansion is mainly driven by an increase in turgor pressure and cell wall loosening^45^. The H^+^-ATPase complex is an H^+^-pump in the plasma membrane that is involved in regulating turgor pressure and cell wall pH^51,52^. In the rosette leaf dataset, we identified four of the 11 H^+^-ATPase gene family members^53^ all of which showed elevated expression in leaves 5 through 8 (Fig. 5b). Although these leaves are still growing, growth here is mainly driven by turgor-mediated cell expansion^50^.

### Developmental stage expression markers

Developmental stages are often characterized by the expression of specific marker genes, like transcription factors which initiate molecular programs at precise times in flower development^54^. Protein synthesis constitutes a time delay for changes in transcript levels to become apparent in protein levels^40^. Stage markers might therefore appear to be out of sync between the proteome and transcriptome datasets in a dynamically developing system like the flower. We compared the expression profiles of a set of gene families that were associated with stage 12 flowers in a transcriptome study by Zhang *et al*.^55^. Oleosins, DUF1216 and DUF220 genes also showed peak mRNA expression at flower stage 11–12 (oleosins, DUF220) and stage 12 (DUF1216) in our transcriptome data (Fig. 5c). At the protein level, however, elevated expression was apparent only at stage 12 (oleosins, DUF220) and stage 13 (DUF1216) (Fig. 5c).

### Phosphorylation site characterization

The number of phosphorylation sites identified for each protein ranged from a single site to more than 30 distinct modification sites. A high number of phosphorylation sites was often detected for proteins with large unstructured domains like loops and tails^56^. For the protein IQD14, which belongs to the family of plant-specific IQ67 Domain (IQD) genes involved in calcium regulation^57^, we detected 28 distinct phosphorylation sites, most of which localized to unstructured regions (Fig. 5d). The expression profiles of these sites in the silique and seed dataset mostly resembled the profiles detected at the protein and transcript level, notably a decreasing abundance during silique growth and an increase throughout the seed development stages (Fig. 5d). This indicates that phosphorylation of these sites is constitutive rather than regulatory^58^. In contrast, a divergent phosphorylation pattern was detected for threonine 271 (T271), which showed increased phosphorylation during silique growth. Similar observations were made for serine 439 and 504 (S439, S504) with a peak in phosphorylation signal at embryo stage 8 (Fig. 5d). These sites might therefore be involved in growth stage dependent regulation of protein function.

## Supplementary information

Supplemental Table S1

Supplemental Table S2

Supplemental Table S3

## Data Availability

Source code used for RNAseq data processing and pre-processing of transcript, protein and phosphorylation site data files is available in GitHub^59^.
